# Retrocecal hernia: A case report

**DOI:** 10.1016/j.amsu.2021.102390

**Published:** 2021-05-12

**Authors:** Amine Fatine, Mounir Bouali, Abdelilah El Bakouri, khalid ElHattabi, Fatimazahra Bensardi, Abdelaziz Fadil

**Affiliations:** a Visceral Surgery Emergency Department, University Hospital Center Ibn Rochd, Casablanca, Morocco; b Faculty of Medecine and Pharmacy, Hassan II University, Casablanca, Morocco

**Keywords:** Retrocecal hernia, Pericecal hernia, Internal hernia, Ileum

## Abstract

**Introduction:**

Retroperitoneal Retrocecal hernias are a rare variety of internal hernias and represent an unusual cause of bowel obstruction. Early diagnosis is based on CT scan and requires knowledge of the pathology in order to avoid small bowel resection. We report a case of retrocecal hernia treated surgically and review the characteristics and treatment of retrocecal hernias in the literature.

**Materials and methods:**

Our work is a retrospective case report with a descriptive aim concerning a patient operated for retrocecal hernia within the department of general surgery of CHU Ibn Rochd Casablanca.

**Case report:**

A 72-year-old man presented to the emergency department with abdominal pain and vomiting that have been evolving for 9 days complicated by an occlusive syndrome 36 hours before the admission. The patient was apyretic, and the abdominal examination noted abdominal meteorism predominantly in the right iliac fossa, absence of abdominal scarring, and free hernial orifices. The abdominal X-ray showed air-fluid levels and the abdominopelvic CT scan found clumping of the dilated small intestines posteriorly and below the cecum. The diagnosis of retrocecal hernia was suspected and the patient was taken to the operating room. The operation was performed by laparotomy through a midline incision. On exploration, the cecum and ascending colon were pushed forward and viable bowel loops were incarcerated in a fossa located posteriorly and below the cecum. The procedure consisted of a collapse of the retrocecal ligaments by right coloparietal collapse.

**Conclusion:**

A bowel obstruction in an apyretic patient without abdominal scarring or parietal hernia should suggest the diagnosis of internal hernia, which must be investigated.

## Highlights

• Retrocecal hernias are a rare variety of internal hernias and represent an unusual cause of bowel obstruction.
• Early diagnosis is based on CT scan and requires knowledge of the pathology.
• The procedure consisted of a collapse of the retrocecal ligaments by right coloparietal collapse.
• We report the case of a retrocecal hernia in an elderly man and describe the anatomy and the characteristic features of this pathology.

## 1 Introduction

Internal hernias represent an uncommon cause of small bowel obstruction that may be increasing in frequency. Because the clinical diagnosis of these hernias is difficult, imaging studies such as computed tomography (CT) and small bowel follow through play an important role.

Retrocecal hernia, a type of internal hernia, is rare. It results in strangulation ileus and, therefore, often requires emergency surgery. Early preoperative diagnosis of pericecal hernias is important to avoid small bowel resection. We report a case of retrocecal hernia treated surgically and review the characteristics and treatment of retrocecal hernias in the literature.the work has been reported in line with the SCARE criteria [1].

## 2 Case report

A 72-year-old man, with no pathological antecedent, presented to the emergency department with abdominal pain and vomiting that have been evolving for 9 days complicated by an occlusive syndrome 36 hours before the admission. The patient was apyretic, and the abdominal examination noted abdominal meteorism predominantly in the right iliac fossa, absence of abdominal scarring, and free hernial orifices.

**Fig. 1:**
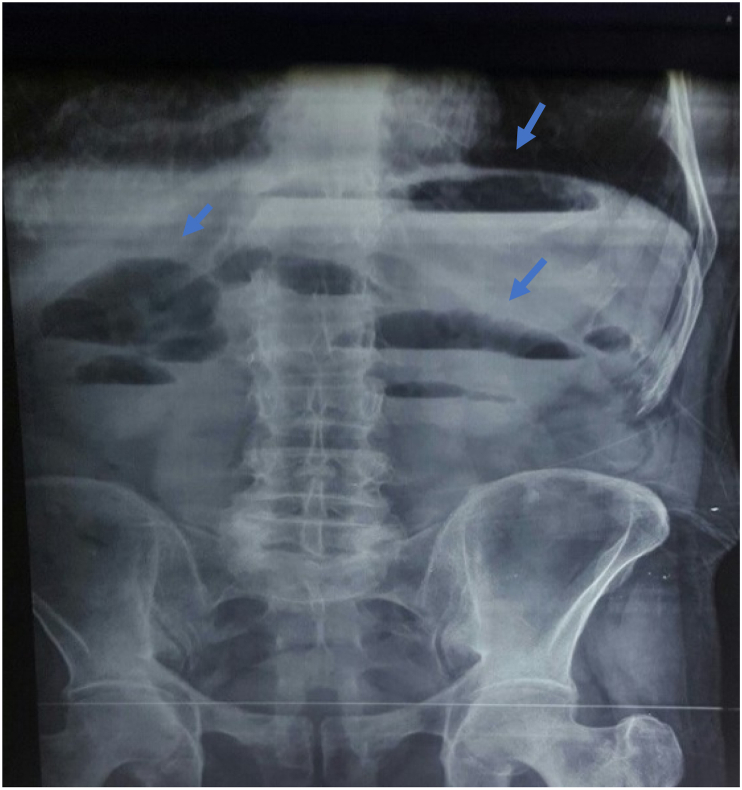
Abdominal X-ray showing air-fluid levels (arrows).

**Fig. 2:**
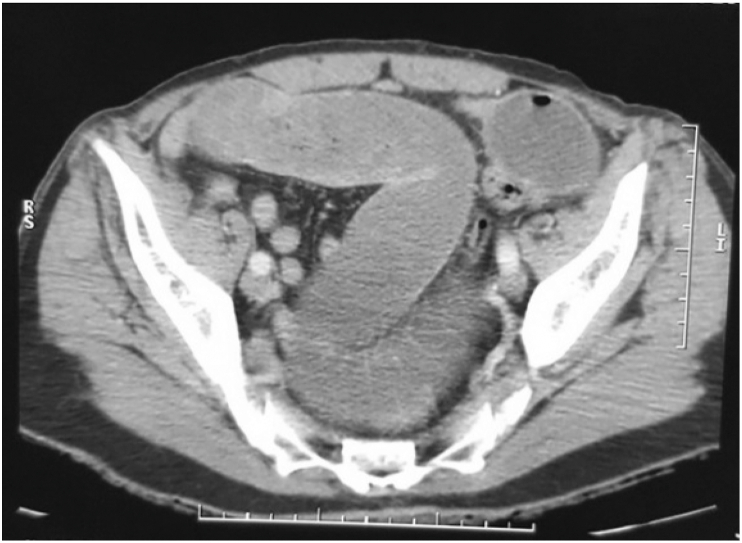
CT scan section showing the retrocecal location of the distal ileum.

The abdominal X-ray showed air-fluid levels and the abdominopelvic CT scan found clumping of the dilated small intestines posteriorly and below the cecum (Fig. 1, Fig. 2).

**Fig. 3:**
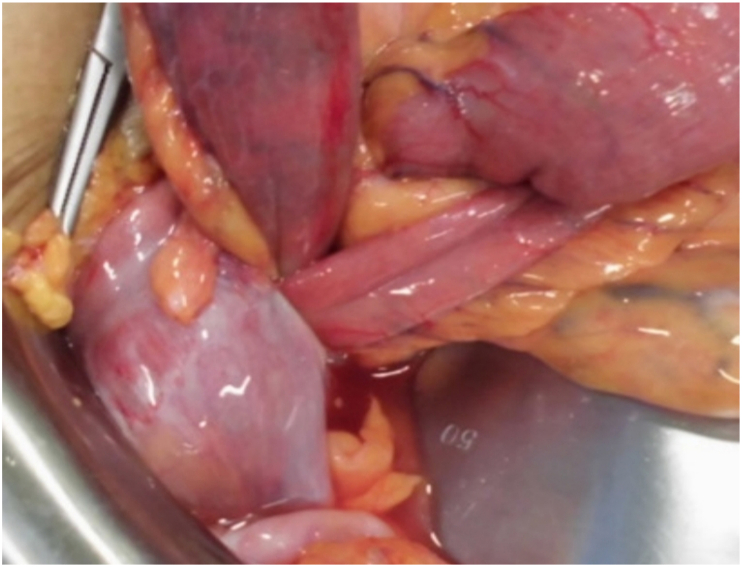
Intraoperative image.

**Fig. 4:**
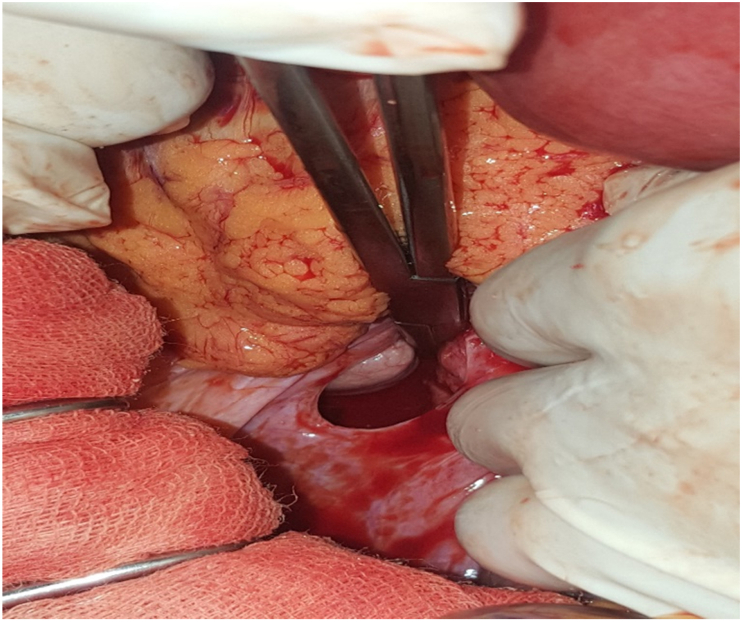
Macroscopic view: intraoperative findings showing the retrocecal fossa.

The diagnosis of retrocecal hernia was suspected and the patient was taken to the operating room. The operation was performed by laparotomy through a midline incision. On exploration, the cecum and ascending colon were pushed forward and viable bowel loops were incarcerated in a fossa located posteriorly and below the cecum (Fig. 3). The neck of the hernia was 3 cm(Fig. 4). The procedure consisted of a collapse of the retrocecal ligaments by right coloparietal collapse.

## 3 Discussion

Internal hernia is defined as the protrusion of a viscera, most commonly the small intestine, through a peritoneal or mesenteric opening, resulting in encapsulation in another compartment [2]. Internal hernias have an autopsy incidence of 0.2–0.9% and represent 0.6–5.8% of the causes of small bowel obstruction [2,3].

There are several types of internal hernias, such as those described by Meyers according to their location, of which para-duodenal hernias are the most frequent [4]. Classically, there are four types of pericecal hernias corresponding to the four peritoneal recesses: the superior and inferior ileocecal recesses, medial to the cecum; the paracolici recesses which are inconsistent and medial to the cecum; and the retrocecal recess (the largest) which is delimited laterally by two posterior peritoneal folds [5].

Generally, these hernias are composed of the terminal ileum and can go up to the right kidney or to the duodenum, pushing forward the cecum or the right colon. They are therefore retroperitoneal hernias.

Clinically, pericecal hernias are manifested by symptoms of acute bowel obstruction. Manifestations may range from nonspecific symptoms such as abdominal distension and nausea to acute abdomen and are proportional to the severity of the obstruction. Internal hernias are initially reducible in most cases and symptoms are usually intermittent, which may cause diagnostic delay leading to significant intestinal suffering [2,5,6]. Thus, the diagnosis should be suspected in the absence of abdominal scarring and symptomatic parietal hernia.

Large recesses, such as the retrocecal recess, increase the risk of intra-hernial volvulus. The main differential diagnosis, in the presence of abdominal scarring, is occlusion by flange and/or adhesions [7].

In front of the signs of small bowel obstruction, the volumetric CT scan remains the best way to make an early diagnosis of retrocecal hernias. It shows the agglutination of the ileal coils, often dilated, behind the cecum which is pushed forward. There is a closed loop occlusion aspect with sometimes several "Bird's beak signs", a modification of the vessels (congestion, stretching) with convergence of these towards the hernial neck [3,7,8]. Dilatation of the upper small intestine is inconstant. Signs of ischaemia s of the herniated small bowel are visualized on the CT scan with contrast medium injection Radiological diagnosis may be difficult in the absence of signs of small bowel ischaemia or volvulus, or when the pathology is not known, and the diagnosis of occlusion by flange may be wrongly made. However, the intraoperative diagnosis of a retrocecal hernia is easy, the cecum is pushed forward and behind it are found the small intestines embedded in a peritoneal recess. During the operation, the classical approach is a median laparotomy but it can also be laparoscopic [9]. The opening of the hernia neck allows the reduction of the herniated coils by gentle traction. In case of severe intestinal ischaemia, a resection-anastomosis may be necessary. The treatment of the hernial sac depends on its size; when it is a small sac, obliteration by simple stitches can be performed, whereas in the presence of a larger sac, as in the case of retrocecal hernia, collapse by coloparietal detachment in the plane of the right Toldt's fascia is performed [6,10].

## 4 Conclusion

Internal hernia is an increasingly common condition that may be diagnosed confidently with CT in most cases. The radiologist plays an important role in the diagnosis of these hernias and may sometimes be the first to suggest the diagnosis to clinicians but clinical suspicion is highly important to not to miss the diagnosis due to the rarity of this condition.

A bowel obstruction in an apyretic patient without abdominal scarring or parietal hernia should suggest the diagnosis of internal hernia, which must be investigated.

## Author statement

This work was carried out in collaboration among all authors. All authors contributed to the conduct of this work. They also declare that they have read and approved the final version of the manuscript.

## Consent

Written informed consent was obtained from the patient for publication of this case report and accompanying images. A copy of the written consent is available for review by the Editor-in-Chief of this journal on request.

## Ethical approval

I declare on my honor that the ethical approval has been exempted by my establishmen.

## Registration of research studies

None.

## Provenance and peer review

Not commissioned, externally peer-reviewed.

## Sources of funding

None.

## Author contributions

Mounir bouali: writing the paper and operating surgeon

Fatine amine: Corresponding author writing the paper and operating surgeon

Abdelilah elbakouri: writing the paper

Khalid elhattabi: writing the paper

Fatimazahra bensardi: study concept.

Abdelaziz fadil: correction of the paper.

## Guarantor

Dr fatine.

## Declaration of competing interest

The authors declare having no conflicts of interest for this article.
